# A1 PRE-CLINICAL CROHN’S DISEASE MICROBIOME PROMOTES INFLAMMATION IN GNOTOBIOTIC RECIPIENT MICE

**DOI:** 10.1093/jcag/gwad061.001

**Published:** 2024-02-14

**Authors:** C Dang, A Chen-Liaw, A Luchak, I Sandhu, K Mu, M Xue, S Lee, I Mogno, C Streutker, G J Britton, J J Faith, W Turpin, K Croitoru

**Affiliations:** Department of Medicine, University of Toronto, Toronto, ON, Canada; Icahn School of Medicine at Mount Sinai, New York, NY; Department of Medicine, University of Toronto, Toronto, ON, Canada; Lunenfeld-Tanenbaum Research Institute, Toronto, ON, Canada; Department of Medicine, University of Toronto, Toronto, ON, Canada; Lunenfeld-Tanenbaum Research Institute, Toronto, ON, Canada; Lunenfeld-Tanenbaum Research Institute, Toronto, ON, Canada; Icahn School of Medicine at Mount Sinai, New York, NY; Unity Health Toronto, Toronto, ON, Canada; Lunenfeld-Tanenbaum Research Institute, Toronto, ON, Canada; Icahn School of Medicine at Mount Sinai, New York, NY; Lunenfeld-Tanenbaum Research Institute, Toronto, ON, Canada; Department of Medicine, University of Toronto, Toronto, ON, Canada

## Abstract

**Background:**

Crohn’s disease (CD) is a chronic disorder of unknow etiology. We recently demonstrated that microbiome composition is associated with the risk of CD development; however, its potential causal effect remains unknown.

**Aims:**

Given the fact that first-degree relatives (FDRs) that are sibling likely to share similar environmental and genetic makeup, we selected pairs of discordant siblings (one remaining healthy referred to as healthy matched control-HMC, and the other who developed CD later on referred to as pre-CD) to investigate whether the microbiome contributes to or triggers dysregulated immune response by transplanting microbiome from them into germ-free mice (GFM).

**Methods:**

Faecal samples from discordant siblings, recruited as part of the CCC-GEM project, a cohort of prospectively followed healthy first-degree relatives, were transplanted into RAG1^-/-^ GFM before T-cell transfer-induced colitis. Fecal Lipocalin-2 (LCN-2) was measured by ELISA to assess gut inflammation. Body weight was monitored for 6 weeks after T-cell transfer. Intestinal tissue damage was assessed by histology scoring (PMID: 8623920). Pro-inflammatory cytokine gene profile of ileum and colon tissue was measured by qPCR. R-software was used for statistical analysis.

**Results:**

Our results showed that mouse recipients of pre-CD microbiota experienced more weight loss than HMC group, with a greater difference observed in the second week after inducing colitis (p=0.0019). LCN-2 of pre-CD mice was significantly higher after T-cell transfer for 3 weeks. Intriguingly, pre-CD stool recipient mice had higher histological damage score in the ileum (p=0.025). Histological analysis revealed that granulomatous inflammation, mucin depletion, crypt loss were greater in pre-CD group (p≤0.05). Pre-CD stool promoted the expression of selected pro-inflammatory cytokine genes in the ileum with significant increases of *TNFα*, *IL-1β*, *IFNγ*, *IL10* and *TGFβ* compared to mice that received HMC microbiome. Colonic tissue showed significant increases in *TNFα* and *TGFβ* in the mice recipient of the pre-CD microbiome.

**Conclusions:**

These results suggests that pre-CD microbiome can worsen T cell transfer colitis in mice compared to HMC microbiome. This suggests that microbiome or other constituents of stool from pre-CD subjects are major contributors to the onset of disease. Modulating pre-CD microbiome composition could lead to novel methods to prevent CD onset.

**Funding Agencies:**

CCC, CIHRWeston Family Foundation, Helmsley, Biocodex

